# Analysis of Bacterial Community Composition of Corroded Steel Immersed in Sanya and Xiamen Seawaters in China via Method of Illumina MiSeq Sequencing

**DOI:** 10.3389/fmicb.2017.01737

**Published:** 2017-09-12

**Authors:** Xiaohong Li, Jizhou Duan, Hui Xiao, Yongqian Li, Haixia Liu, Fang Guan, Xiaofan Zhai

**Affiliations:** ^1^Key Laboratory of Marine Environmental Corrosion and Biofouling, Institute of Oceanology, Chinese Academy of Sciences Qingdao, China; ^2^College of Marine Life Sciences, Ocean University of China Qingdao, China

**Keywords:** bacterial community, MIC, carbon steel, Illumina MiSeq sequencing, 16S rRNA gene

## Abstract

Metal corrosion is of worldwide concern because it is the cause of major economic losses, and because it creates significant safety issues. The mechanism of the corrosion process, as influenced by bacteria, has been studied extensively. However, the bacterial communities that create the biofilms that form on metals are complicated, and have not been well studied. This is why we sought to analyze the composition of bacterial communities living on steel structures, together with the influence of ecological factors on these communities. The corrosion samples were collected from rust layers on steel plates that were immersed in seawater for two different periods at Sanya and Xiamen, China. We analyzed the bacterial communities on the samples by targeted 16S rRNA gene (V3–V4 region) sequencing using the Illumina MiSeq. Phylogenetic analysis revealed that the bacteria fell into 13 phylotypes (similarity level = 97%). *Proteobacteria*, *Firmicutes* and *Bacteroidetes* were the dominant phyla, accounting for 88.84% of the total. *Deltaproteobacteria*, *Clostridia* and *Gammaproteobacteria* were the dominant classes, and accounted for 70.90% of the total. *Desulfovibrio* spp., *Desulfobacter* spp. and *Desulfotomaculum* spp. were the dominant genera and accounted for 45.87% of the total. These genera are sulfate-reducing bacteria that are known to corrode steel. Bacterial diversity on the 6 months immersion samples was much higher than that of the samples that had been immersed for 8 years (*P* < 0.001, Student’s *t*-test). The average complexity of the biofilms from the 8-years immersion samples from Sanya was greater than those from Xiamen, but not significantly so (*P* > 0.05, Student’s *t*-test). Overall, the data showed that the rust layers on the steel plates carried many bacterial species. The bacterial community composition was influenced by the immersion time. The results of our study will be of benefit to the further studies of bacterial corrosion mechanisms and corrosion resistance.

## Introduction

Structural steel is widely used in marine environments because it is strong, readily available, easy to fabricate, and cost-effective, overall. However, steel is subject to corrosion. This is a serious worldwide problem and has a great social and economic impact (Hou et al., 2017). Corrosion is caused by complex chemical, physical and biological processes (Kip and Veen, 2015). Biological (in fact, microbiological influenced corrosion MIC) plays a critical role (Baboian, 2005). MIC is caused by electrochemical reactions created by those microorganisms that form ‘biofilms’ on immersed steel structures (Hamilton, 1991). Fungi are closely associated to this process (e.g., *Arthrinium phaeospermum*, *Aspergillus niger*, *Chrysosporium merdarium* and acidotolerant black yeast) (Lugauskas et al., 2009; Leo et al., 2013). Lugauskas et al. (2009) found that various strains of the same fungal species have different influences on submerged metal surfaces. However, bacteria are the main component of the biofilms, and contribute most to MIC (Bermont-Bouis et al., 2007) and the formation and transformation of corrosion products (Sun H. et al., 2014). The metabolic activities of bacterial communities within the biofilms interact with environmental factors, such as dissolved oxygen, pH, organic, and inorganic compounds, etc., to influence the electrochemical state of the metal and influence the rate of corrosion (Beech, 2004; Beech and Sunner, 2004; Coetser and Cloete, 2005; Videla and Herrera, 2005). It is also known that the bacterial surface associations within biofilms influence the electrochemical reaction rate (Dang and Lovell, 2016). Diverse bacterial populations can coexist in biofilms and often form synergistic communities (consortia) which contribute to the electrochemical processes via cooperative metabolic processes (Gonzalez-Rodrıguez et al., 2008; Korenblum et al., 2008).

Some of the bacteria species that are associated with steel corrosion have been identified. They includes sulphate-reducing bacteria (SRB), sulphur-oxidizing bacteria (SOB), iron-reducing bacteria (IRB), and iron-oxidizing bacteria (IOB) (Sun J. et al., 2014), etc. SRB are regarded as the most influential (Duan et al., 2008), and are regarded as the main corrosion-accelerating factor in the context of the MIC of metals in marine environments (Angell and Urbanic, 2000). Other types of bacteria may also play an important role, e.g., methanogens and metal reducing-bacteria (Zhu et al., 2003; Gonzalez-Rodrıguez et al., 2008). Moreover, what is interesting is that bacteria not only cause corrosion but can also inhibit or protect against corrosion, which is termed as MIC inhibition (MICI) (Zuo, 2007). There is currently a focus on exploiting bacteria and their metabolic by-products, including biofilm and extracellular polymeric substances (EPSs), to reduce MIC. The aim is to replace the biocides and toxic evaporative, organic compounds that are currently employed as rust retardants (Grooters et al., 2007). The mechanisms of MIC and MICI are not completely understood. They cannot be connected with a single biochemical reaction or a single bacterial species or cluster (Kip and Veen, 2015). It is therefore necessary, in this context, to learn more about the nature of the species complexes that form on corroding steel and rust that is immersed in seawater, so as to learn how to protect steel structures in marine environments.

Analyses of the bacterial communities of early developing biofilms in the rust layers of steel originally relied upon plate culturing techniques (Bermont-Bouis et al., 2007), which is laborious, imprecise, and time-consuming. Significantly, nearly all of the bacterial species from this environment do not reproduce on culture plates (Dunbar et al., 1999). Advances in molecular biology now permit us to analyze bacterial communities with considerable more precision. The techniques we adopted to investigate the composition of the bacterial communities were terminal restriction fragment length polymorphism (T-RFLP), denaturant gradient gel electrophoresis (DGGE), fluorescence *in situ* hybridization (FISH), and 16S rRNA gene libraries. *Proteobacteria* was recognized as the dominant bacterial group during the first 36 h of biofilm formation by using 16S rRNA gene libraries and T-RFLP (Lee et al., 2008). *Citrobacter* spp., *Enterobacter* spp. and *Halanaerobium* spp. were identified as the dominant bacteria of biofilms after 40-days immersion by ribosomal library and DGGE. FISH analysis was also used in the study of bacterial community composition, and the results showed that *Alphaproteobacteria* was the dominant community during the first few weeks of biofilm growth. In addition, it became apparent that the combination of FISH and confocal microscopy was of critical importance. It allowed us to define the relative importance of different bacteria in causing corrosion, and provided information both about the spatial structure of the corrosion biofilms, and quantitative information about the bacteria (Dang and Lovell, 2002a,b). Recently, high-throughput Illumina sequencing has been frequently used to investigate the bacterial community composition of various environments (Moreau et al., 2014; Sun J. et al., 2014; Chao et al., 2015). and allowed us to gain deeper insight into the bacterial community composition of the samples (Bokulich and Mills, 2012; Mayo et al., 2014). Our research was greatly enhanced by access to MiSeq sequencing which allowed us to obtain comprehensive information covering the composition of the bacterial communities we targeted. This follows Vigneron et al. (2016) who adopted this technique to reveal that *Desulfovibrio* species was the dominant bacteria on an offshore oil production facility. We consider that the application of this technology in the current area of research is in its infancy.

In this study we characterized the composition of the bacterial communities in corrosion samples that had been collected from rust layers on steel plates that had been immersed in seawater, by means of high-throughput Illumina MiSeq sequencing. In addition, we analyzed the influence of ecological factors on the bacterial communities. The results of our study have important implications for further study of bacterial corrosion mechanisms and anti-corrosion.

## Materials and Methods

### Sample Sites and Collection

The plates of steel had the following composition (wt. %): C 0.16, Si 0.12, Mn 0.45, S 0.029, and P 0.019. Nine samples were collected in December 2014 for this study. Among them, six samples (SE1, SE2, SE3, SE4, SE5, and SOH) were collected from the coastal zone of the Hongtang Bay which is located in Sanya City, Hainan Province. The sample identified as SOH provided us with rust layers from steel plates that had been immersed in seawater for 6 months. Samples identified as XE4, XE5 and XE6 were collected from the rust layers of steel plates that had been immersed in seawater for 8 years in a coastal zone of the island of Gulang, which is situated in Xiamen City, Fujian Province.

Large fouling organisms were removed with sterile forceps in sterile conditions from the steel plates as soon as they were removed from the sea. The surface of the test material was gently rinsed in sterilized seawater to remove unattached bacteria. The deposits were sampled with metallic spatulas, taking care not to crush the samples or expose them to air for too long. They were immediately placed in 10 ml sterile plastic centrifuge tubes, transported to the laboratory on dry ice, and were stored at -80°C pending analysis (Païssé et al., 2013). Meanwhile, the salinity, temperature and pH of the seawater were measured by multiparameter water quality detector (CTD90M, Germany).

### DNA Extraction

The total community genomic DNA of each sample was extracted according to the method of Zhou et al. (1996). Five microliter of each genomic DNA were subjected to 1% agarose gel electrophoresis to examine its integrity. The concentration of the DNA was measured with a UV-vis spectrophotometer (NanoDrop 2000c, United States) to identify that adequate amounts of high-quality total genomic DNA were extracted.

### 16S rRNA Gene Amplification by PCR

V3–V4 region of the bacterial 16S rRNA gene was amplified by PCR (95°C for 3 min followed by 27 cycles of 95°C for 30 s, 55°C for 30 s and 72°C for 45 s and a final extension at 72°C for 10 min using the primers 338F 5′-barcode-ACTCCTACGGGAGGCAGCAG-3′ and 806R 5′-GGACTACHVGGGTWTCTAAT-3′ (Dennis et al., 2013), where the barcode was an eight-base sequence that was unique to each sample. The PCR reactions were performed in triplicate in 20 μl reactions, containing 2 μl of 10× Ex Taq buffer, 2 μl of 2.5 mM dNTPs, 0.8 μl of each primer (5 μM), 0.2 μl Ex Taq polymerase, 0.2 μl of BSA, 14 μl of ddH_2_O and 10 ng of template DNA.

### Illumina MiSeq Sequencing

The amplicons were extracted from 2% agarose gels and purified using the AxyPrep DNA Gel Extraction Kit (Axygen Biosciences, Union City, CA, United States) according to the manufacturer’s instructions. The purified amplicons were quantified using QuantiFluor^TM^ -ST (Promega, United States), pooled in equimolar ratios and subjected to paired-end sequencing (2 × 250) on an Illumina Miseq platform according to standard protocols. The raw reads were deposited into the NCBI Sequence Read Archive (SRA) database.

### Processing Sequencing Data

The raw fastq files were demultiplexed and quality-filtered using QIIME (version 1.9.1) (Caporaso et al., 2010) with the following criteria: (i) The output data (reads) were truncated at any site receiving an average quality score < 20 over a 50 base pair (bp) sliding window. (ii) Primers were matched exactly allowing a two nucleotide mismatching, and reads containing ambiguous bases were removed. (iii) Sequences whose overlap was longer than 10 bp were merged according to their overlap sequence. Operational taxonomic units (OTUs) were clustered with a 97% similarity cut-off using UPARSE version 7.1^1^ (Edgar, 2013). The normalization process followed OTU clustering. Chimeric sequences were identified and removed using UCHIME (Edgar, 2010; Edgar et al., 2011). The taxonomy of each 16S rRNA gene sequence was analyzed with RDP Classifier^2^ (Wang et al., 2007) against the Silva (SSU128) 16S rRNA database using a confidence threshold of 70% (Quast et al., 2013).

The relative abundances of the phylum, class and genus levels were plotted as a bar graph. Heatmaps based on the relative abundance of OTUs at the phylum and genus levels were also generated with R program (R Development Core Team, 2013). A venn diagram was created using Mothur v.1.30.1 (Schloss et al., 2009) to identify the similarities and differences of the communities in the three kinds of samples (sample SOH, samples from Sanya, and samples from Xiamen). In alpha diversity analysis, alpha diversity parameters such as Chao, Ace, Simpson, and Shannon were estimated using mothur (version v.1.30.1^3^) with a 97% similarity cut-off (Schloss et al., 2009). They provided a means of evaluating the potential total number of OTU and an estimate of the level of diversity in each sample. Rarefaction curves based on these metrics were generated. In beta diversity analysis, differences in the bacterial communities among the nine samples were preformed by a hierarchical cluster tree created using the unweighted pair-group method with arithmetic mean (UPGMA). A principal co-ordinates analysis (PCoA) plot was also obtained using Mothur with the calculation of Bray–Curtis (Schloss et al., 2009).

### Data Accession Number

The obtained raw sequences were deposited in the NCBI database (Accession Number: PRJNA396473).

## Results

### Diversity Analysis and Richness of OTUs

**FIGURE 1:**
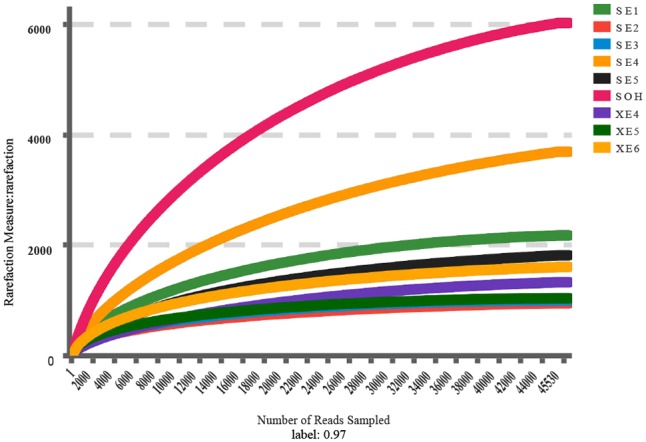
Rarefaction analysis of the V3/V4 MiSeq sequencing reads of the 16S rRNA gene from the nine corrosion samples at a 97% sequence similarity cutoff value.

A total 558,632 high-quality bacterial V3–V4 Illumina sequences, ranging from 47,920 to 77,230, were obtained for further analysis (**Table 1**). Data were normalized by subsampling the 16S rDNA data at 45,530 reads per sample to correct for unequal sequencing depth. The average length of the high-quality sequences from the nine corrosion samples was 441 bp. After random re-sampling at the 0.03 distance level, the average number of OTUs in the 8 years samples was 1,695. However, there were 6,020 OTUs in the sample immersed for 6 months. For the 8 years samples the average numbers were: OTUs 1,695, ACE 1,955 Chao1 index 1826, Shannon and Simpson index 4.70 and 0.0378. For the 6 months sample the numbers were of OTUs 6,020, ACE 7,026 and Chao1 index 6,422, Shannon and Simpson index 7.11 and 0.0050 (**Table 1**). The rarified Chao1 and Shannon diversity indexes showed remarkable differences across the 8 years samples and the 6 months sample (*P* < 0.001, Student’s *t*-test). Bacterial diversity and richness were higher in the 6 months immersion sample compared to the 8 years immersion samples, as described by the Shannon and Simpson diversity indices, ACE and Chao1 index. This was also confirmed by rarefaction curve analyses of the OTUs (**Figure 1**). Meanwhile, the average richness of 8 years immersion samples from Sanya (ACE index 2,249 and Chao1 index 2,092) was higher than that from Xiamen (ACE index 1,463 and Chao1 index 1,383). However, both of the rarified Chao1 and Shannon diversity indexes were not significantly different (*P* > 0.05, Student’s *t*-test) across the 8 years samples from Sanya and Xiamen.

**Table 1:** Number of sequences analyzed, OTUs, estimated community richness indices (Chao and Ace), coverage, and community diversity indices (Shannon and Simpson) of the 16S rRNA libraries of the corrosion samples.

Sample ID	Reads	0.97					
		Sobs	Ace	Chao	Coverage	Shannon	Simpson
SE1	56978	2171	2323	2212	0.9940	5.41	0.0145
SE2	61006	943	1034	978	0.9969	4.21	0.0430
SE3	47920	993	993	993	1.0000	4.45	0.0273
SE4	75126	3687	4747	4238	0.9748	5.91	0.0115
SE5	77230	1811	2150	2038	0.9901	4.03	0.0924
SOH	64486	6020	7026	6422	0.9701	7.11	0.0050
XE4	59698	1323	1502	1384	0.9942	3.97	0.0511
XE5	50966	1033	1057	1036	0.9989	4.84	0.0196
XE6	65222	1600	1830	1728	0.9930	4.81	0.0431


**FIGURE 2:**
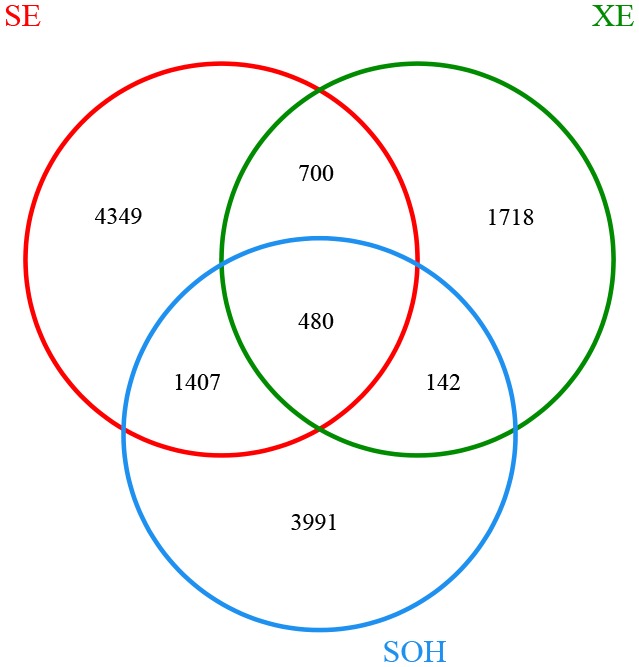
Venn diagram was at a distance of 0.03. There were 6,936 OTUs in sample SE (five corrosion samples that were collected from Sanya and had been immersed in seawater for 8 years). There were 3,040 OTUs in sample XE (three corrosion samples that were collected from Xiamen and had been immersed in seawater for 8 years). There were 6,020 OTUs in sample SOH.

The Venn diagram showed that SE (8 years immersion samples from Sanya) and XE (8 years immersion samples from Xiamen) shared 1,180 OTUs, SE and SOH shared 1,887 OTUs, XE and SOH shared 622 OTUs and 480 OTUs were shared by all nine samples (**Figure 2**). The average number of OTUs in the 8 years samples from Sanya and Xiamen were 1,387 and 1,013, respectively. In addition, the Good’s coverage values (**Table 1**) and the rarefaction curves of all corrosion samples (**Figure 1**) indicated that the 16S rRNA gene sequences derived from these corrosion samples could represent the total bacterial community in this study.

### Analysis of Bacterial Communities

**FIGURE 3:**
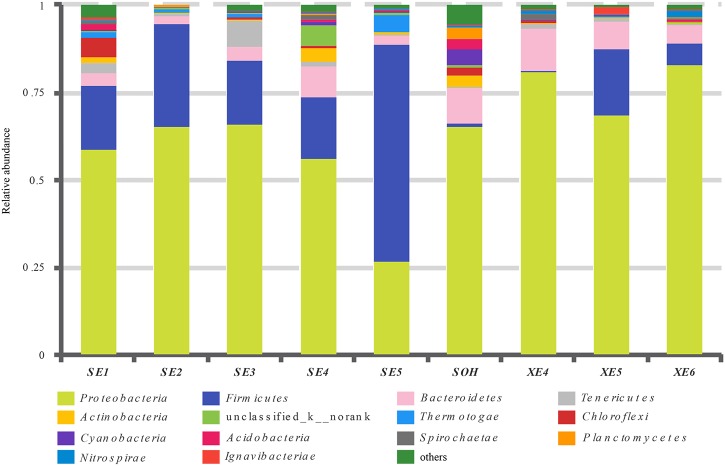
Relative abundances of bacterial 16S rRNA gene sequences from the corrosion samples presented at the phylum level.

**FIGURE 4:**
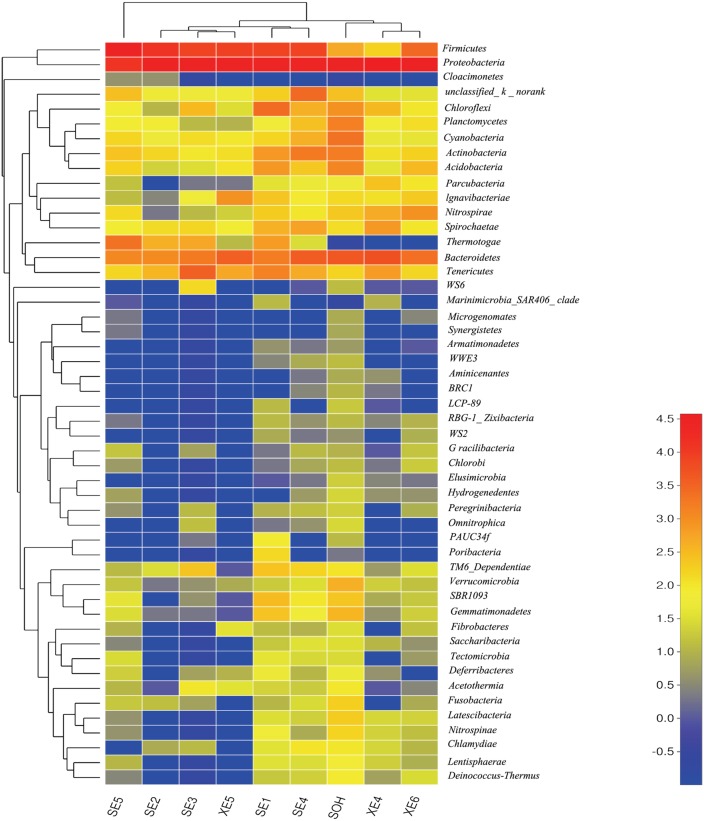
The bacterial community distributions among the nine corrosion samples at the phylum level.

At the phylum level, more than 13 prokaryotic phyla were found in the nine samples accounting for 95.35% of the total community, namely *Proteobacteria* (63.44%), *Firmicutes* (19.12%), *Bacteroidetes* (6.28%), *Tenericutes* (1.57%), *Actinobacteria* (0.99%), *Chloroflexi* (0.86%), *Thermotogae* (0.81%), *Cyanobacteria* (0.54%), *Acidobacteria* (0.49%), *Planctomycetes* (0.36%), *Spirochaetae* (0.32%), *Nitrospirae* (0.34%) and *lgnavibacteriae* (0.23%) (**Figure 3** and **Table 2**). *Proteobacteria*, *Firmicutes* and *Bacteroidetes* were the core phyla, accounting for nearly 88.84% of the total. For the majority of corrosion samples, *Proteobacteria* was the dominant phylum, ranging from 26.82 to 82.93% of the total number of phyla. *Firmicutes* was the second most represented phylum, ranging from 0 to 62.14% of the total number of phyla. *Bacteroidetes* was the third most dominant phylum, ranging from 2.39 to 12.00% of the total number of phyla. However, in SE5, *Firmicutes* (62.14%) and *Proteobacteria* (26.82%) were the first and second most abundant phyla, which was markedly different to the distribution in the other samples. The remaining 10 phyla were represented at a low level on individual samples. Furthermore, the hierarchical clustering heat map of the in-depth taxonomic analysis was plotted to compare the membership and structure of each sample at the phylum level. It also indicated that *Proteobacteria*, *Bacteroidetes* and *Firmicutes* were the three dominant bacterial communities (**Figure 4**).

**Table 2:** Microbial community compositions at phylum level.

	Total taxonomy (%)	SE1(%)	SE2(%)	SE3(%)	SE4(%)	SE5(%)	SOH(%)	XE4(%)	XE5(%)	XE6(%)
*Proteobacteria*	63.44	58.74	65.31	65.99	56.18	26.82	65.45	80.96	68.54	82.93
*Firmicutes*	19.12	18.40	29.38	18.26	17.80	62.14	1.04	0	18.92	6.17
*Bacteroidetes*	6.28	3.45	2.39	4.03	8.84	2.61	9.95	12.00	8.00	5.24
*Tenericutes*	1.57	3.05	0	7.56	1.01	0	0	1.46	1.08	0
*Actinobacteria*	0.99	1.57	0	0	3.91	0	3.44	0	0	0
*Chloroflexi*	0.86	5.67	0	0	0	0	2.03	0	0	0
*Thermotogae*	0.81	1.46	0	1.08	0	4.78	0	0	0	0
*Cyanobacteria*	0.54	0	0	0	0	0	4.85	0	0	0
*Acidobacteria*	0.49	1.70	0	0	0	0	2.68	0	0	0
*Planctomycetes*	0.36	0	0	0	0	0	3.23	0	0	0
*Spirochaetae*	0.32	0	0	0	1.29	0	0	1.59	0	0
*Nitrospirae*	0.34	0	0	0	0	0	0	1.00	0	2.04
*lgnavibacteriae*	0.23	0	0	0	0	0	0	0	2.07	0
Others	4.65	5.96	2.92	3.08	10.97	3.65	7.33	2.99	1.39	3.62


**FIGURE 5:**
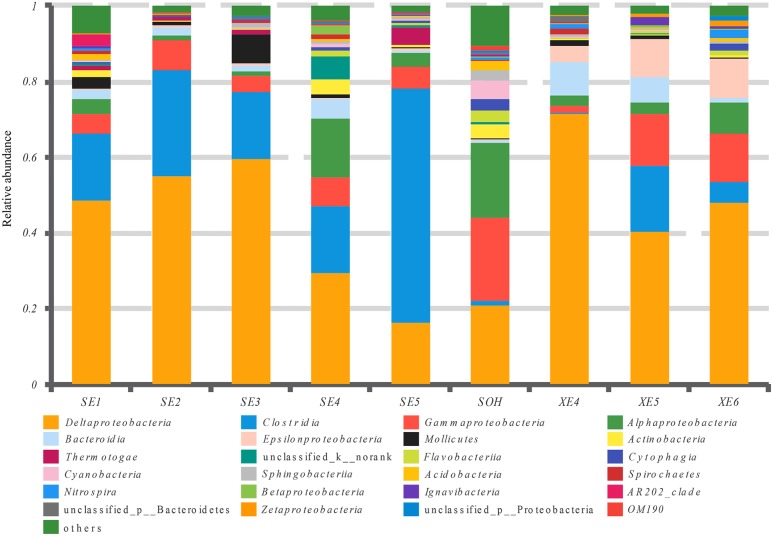
Relative abundances of bacterial 16S rRNA gene sequences from the corrosion samples presented at the class level.

**FIGURE 6:**
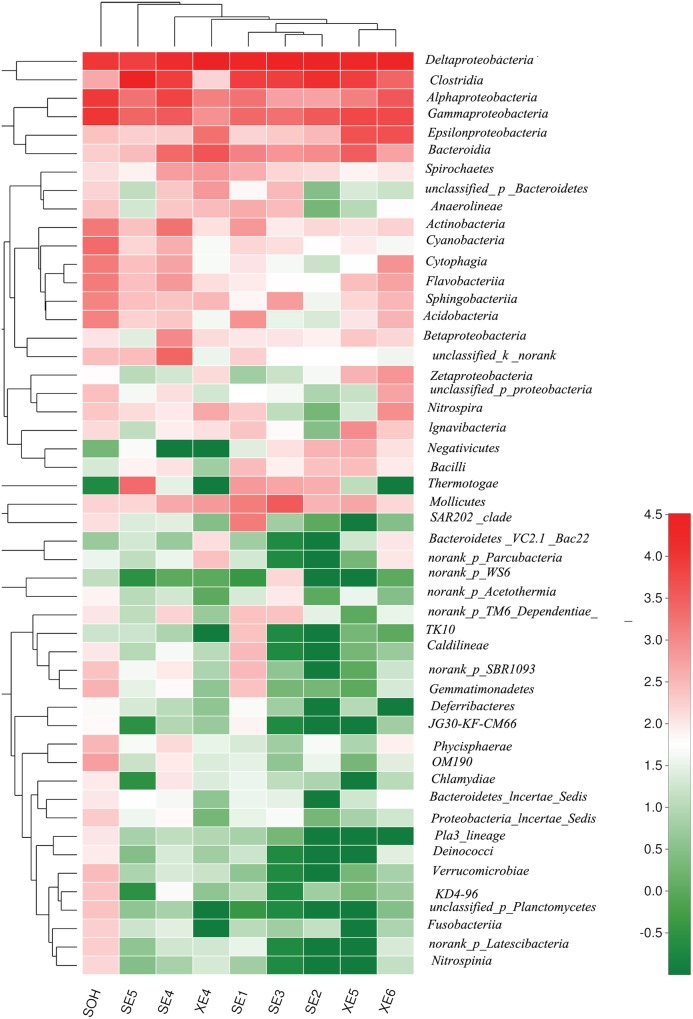
The bacterial community distributions among the nine corrosion samples at the class level.

At the class level, more than 21 classes of prokaryote were found overall and accounted for 86.80% of the total community (**Figure 5** and **Table 3**). For the majority of corrosion samples*, Deltaproteobacteria* was the most abundant class, ranging from 16.50 to 71.56% according to the samples. *Clostridia* came second and ranged from 0 to 61.85% of the whole community. *Gammaproteobacteria* was the third most dominant class, ranging from 1.78 to 22.0% of the whole bacterial community. Some other classes (e.g., *Alphaproteobacteria* and *Bacteroidia)* also occupied a relatively large proportion of the bacterial community composition, based on the average abundance analysis (**Figure 5** and **Table 3**). In addition, some classes, which occupied a relatively small proportion of the community composition, but which have been associated with corrosion (such as *Zetaproteobacteria*), were also found in this study (**Figure 5**). However, the community composition of some samples was unique at the class level. For example, *Clostridia* (61.85%) and *Deltaproteobacteria* (16.50%) were the first and second dominant classes in SE5. *Bacteroidia* (9.06%) and *Gammaproteobacteria* (12.59%) were the second dominant bacterial class in XE4 and XE6, respectively. *Alphaproteobacteria* was the third dominant bacterial classes in SE4 (15.48%) and SOH (19.91%). Furthermore, the hierarchical clustering heat map was also plotted to compare the membership and structure of each sample at the class level. It also indicated that *Deltaproteobacteria*, *Clostridia* and *Gammaproteobacteria* were the dominant three bacterial communities among the top 50 classes across all the samples (**Figure 6**).

**Table 3:** Microbial community compositions at class level.

	Total taxonomy (%)	SE1(%)	SE2(%)	SE3(%)	SE4(%)	SE5(%)	SOH(%)	XE4(%)	XE5(%)	XE6(%)
*Deltaproteobacteria*	43.48	48.80	55.18	59.70	29.71	16.50	21.18	71.56	40.63	48.08
*Clostridia*	18.44	17.67	28.03	17.83	17.48	61.85	0	0	17.41	5.67
*Gammaproteobacteria*	8.98	5.12	8.01	4.09	7.81	5.73	22.00	1.78	13.72	12.59
*Alphaproteobacteria*	6.63	3.97	1.16	1.24	15.48	3.85	19.91	2.75	2.88	8.39
*Bacteroidia*	3.16	2.55	2.14	1.66	5.03	0	0	9.06	6.81	1.18
*Epsilonproteobacteria*	2.74	0	0	0	0	0	0	4.19	9.98	10.50
*Mollicutes*	1.57	3.05	0	7.56	1.01	0	0	1.46	1.08	0
*Actinobacteria*	0.99	1.57	0	0	3.91	0	3.44	0	0	0
*Thermotogae*	0.81	1.46	0	1.08	0	4.78	0	0	0	0
Others	13.20	15.81	5.48	6.84	19.57	7.29	33.47	9.20	7.49	13.59


**FIGURE 7:**
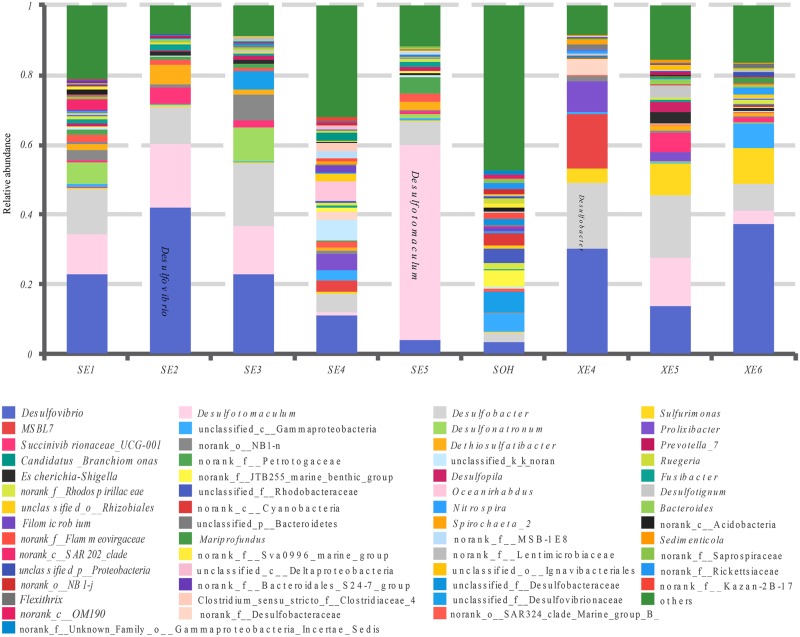
Relative abundances of bacterial 16S rRNA gene sequences from the corrosion samples presented at the genus level.

**FIGURE 8:**
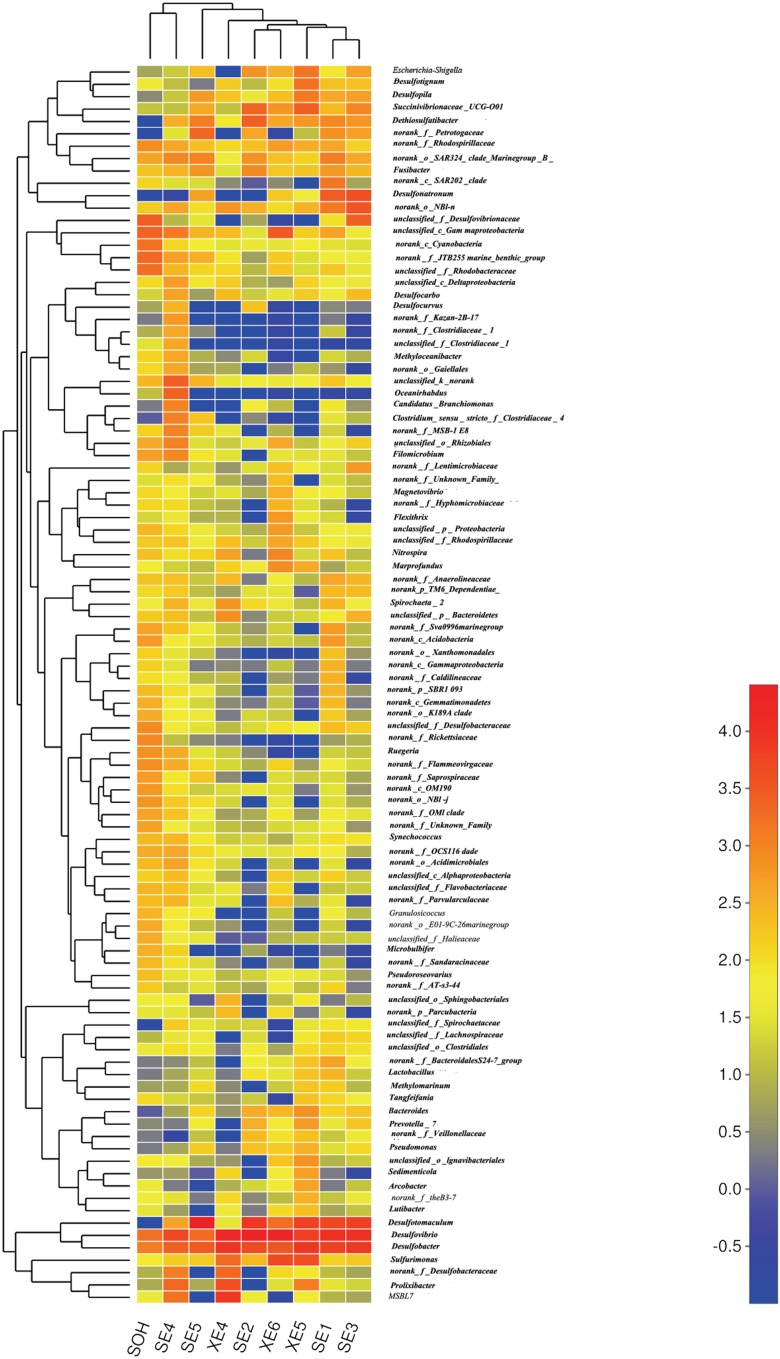
The bacterial community distributions among the nine corrosion samples at the genus level.

More than 56 genera were identified (**Figure 7** and **Table 4**). For the majority of corrosion samples*, Desulfovibrio* was the most abundant, ranging from 3.59 to 42.04% of the total number of genera. *Desulfobacter* came second (2.70–18.75%), and *Desulfotomaculum* was the third (0–56.04%). Other genera were well represented, based on the average abundance analysis (e.g., *Sulfurimonas* and *Desulfonatronum*) (**Figure 7** and **Table 4**). The generic profile of some samples was unique. For example, *Desulfotomaculum* (56.04%) and *Desulfobacter* (18.01%) were the dominant genera in SE5 and XE5, respectively. *Sulfurimonas* (10.31%) was the second most dominant genus in samples XE6. The hierarchical clustering heat map indicated that *Desulfovibrio*, *Desulfobacter* and *Firmicutes* were the dominant three bacterial genera among the top 100 genera (**Figure 8**).

**Table 4:** Microbial community compositions at genus level.

	*Desulfovibrio*	*Desulfobacter*	*Desulfotomaculum*	*Sulfurimonas*	*Desulfonatronum*	Others
SE1(%)	23.04	13.23	11.35	0	6.17	46.21
SE2(%)	42.04	10.69	18.38	0	0	28.89
SE3(%)	23.07	17.86	13.90	0	9.74	35.43
SE4(%)	11.07	5.46	0	0	0	83.47
SE5(%)	4.14	6.76	56.04	0	1.07	31.99
SOH(%)	3.59	2.70	0	0	0	93.71
XE4(%)	30.36	18.75	0	4.17	0	46.72
XE5(%)	13.81	18.01	14.00	8.97	0	45.21
XE6(%)	37.41	7.73	3.95	10.31	0	40.60
Total taxonomy(%)	20.95	11.97	12.95	2.61	1.89	49.63


### Beta Diversity Analysis of the Nine Corrosion Samples

**FIGURE 9:**
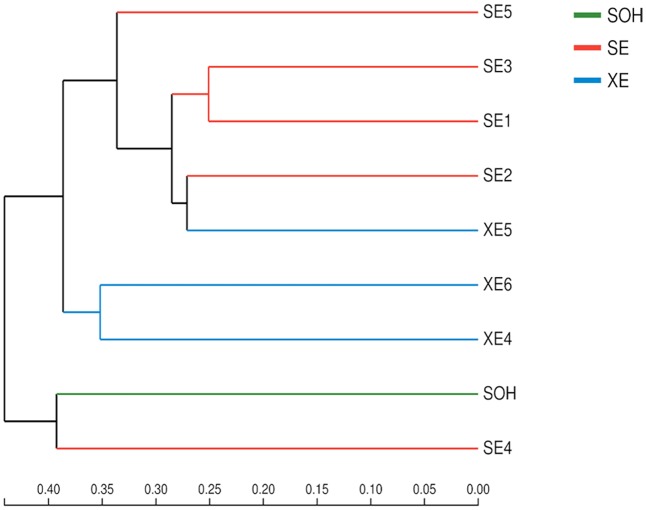
A hierarchical cluster tree created using UPGMA with Bray–Curtis at the level of OTU. Microbial community distribution patterns at a 97%-similarity OTU level.

**FIGURE 10:**
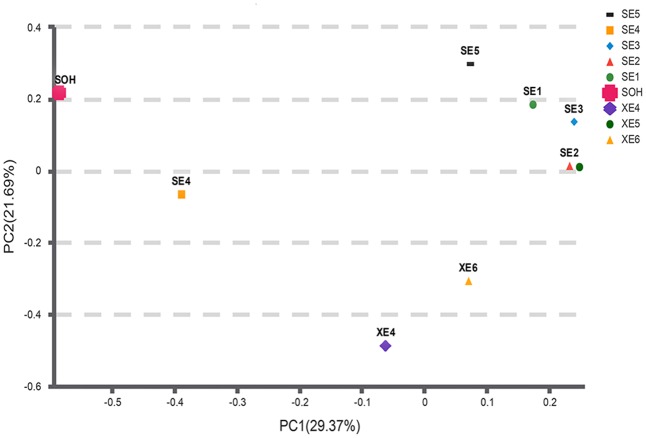
A PCoA scatter plot was calculated with Bray-Curtis. The first two components PC1 and PC2 explain 29.37 and 21.69% of the variations, respectively. Microbial community distribution patterns at a 97%-similarity OTU level.

Two methods were adopted to analyze the beta diversity of the nine samples (**Figures 9, 10**). Firstly, a hierarchical cluster tree of the bacterial communities was constructed by means of the UPGMA at a 97%-similarity OTU level. This showed that the data were clustered in two distinct groups (**Figure 9**). Group 1 contained the 6 months immersion sample (SOH) and one 8 years immersion sample (SE4). Group 2 included the other 8 years immersion samples. Afterwards, a principal coordinates analysis (PCoA) then targeted major bacterial clades, and confirmed the output of the first method, and explained 51.06% of the observed variation (**Figure 10**). Eight years immersion samples (except SE4) were grouped to the right of the graph along PC1. SOH was separated from the 8-years immersion samples and grouped to the left of the graph along PC1. Whereas SE4 was grouped in the middle of the graph between SOH and the other 8-year immersion samples. There was a clear distinction between SOH and the other corrosion samples along the first axis. Furthermore, bacterial communities were separated by the second axis. The results of the two methods indicated that the bacterial diversity (bacterial community composition) was clearly correlated to the immersion period. The sea area had no influence on the composition of the bacterial community.

## Discussion

### Analysis of Composition of the Bacterial Community of the Corrosion Samples

Compared with samples from other environments, such as marine sediments (Liu et al., 2015), and seawater samples (Suh et al., 2015; Yang et al., 2015), the composition of the bacterial communities on the corrosion samples was similar at the phylum level, but significantly different at the genus level. In this study, *Proteobacteria*, *Bacteroidetes* and *Firmicutes* were the three core phyla in all samples. *Proteobacteria* was dominant in the majority of samples. This was also observed by Vigneron et al. (2016). They analyzed the bacterial community composition of corrosion samples taken from an offshore oil production facility. *Proteobacteria* also emerged as the dominant bacterial phylum in the initial stage of biofilm formation on carbon steel (Bermont-Bouis et al., 2007; Jones et al., 2007; Lee et al., 2008; Dang et al., 2011; McBeth and Emerson, 2016). We can point to a number of reasons for the dominance of *Proteobacteria* in rust samples. Members of this phylum are pioneer surface colonizers and important biofilm ‘builders.’ The ‘facilitation’ of biofilm formation is an important step in the further development of diverse populations, and their on-going stability (Slightom and Buchan, 2009; Dang et al., 2008, 2011). It is noteworthy that *Proteobacteria* is also the largest bacterial phylum and the most abundant across a range of environmental conditions (Liu et al., 2015; Qi et al., 2016) and in seawater (Suh et al., 2015; Yang et al., 2015; Mancuso et al., 2016).

*Bacteroidetes* was the second most abundant phylum. It was found to be dominant in biofilms formed on steel plates immersed in the sea for 40 days (Dang et al., 2011; McBeth and Emerson, 2016). *Bacteroidetes* is a dominant phylum in marine environments (Kirchman, 2002), and has also been linked to biological corrosion. *Bacteroidetes* can also contribute to the survival of other of surface colonizers and the formation and development of biofilms (Dang et al., 2011). The composition and abundance of surface-associated bacterial colonies may be influenced by ‘predatory’ members of the phylum *Bacteroidetes* (Dang and Lovell, 2016). *Bacteroidetes* members are known to degrade complex biopolymers (Kirchman, 2002), which may assist in the creation of an aerobic environment with a biofilm, that is conducive to the growth of colonizing bacteria. *Firmicutes* was the third most dominant phylum in the majority of corrosion samples. It was found to be abundant in biofilms in the rust layer, based on 16S rRNA gene (Zhang and Fang, 2001; Luan et al., 2012). The presence of a *Firmicutes* member, *Tindallia texcoconensis*, isolated from lake Texcoco, Mexico by Alazard et al. (2007), was associated with hydrogen production, that provided for SRB. Some members of this phylum generate H_2_S and organic acids that can cause corrosion. For example, *Acetobacterium carbinolicum* produces acetic acid which can corrode steel (Paarup et al., 2006).

At the class level, *Deltaproteobacteria* was the dominant class in the majority of corrosion samples. This observation parallels information from studies of the bacterial communities of samples collected from water-flooded petroleum reservoirs, water injection systems of Brazilian offshore oil platforms, and corrosive petroleum reservoirs in Yangzhou (Korenblum et al., 2010; Li et al., 2016; Tian et al., 2017). There are many important SRB groups belonging to this class, e.g., *Desulfovibrio*, *Desulfobacter* and *Desulfonatronum*. Some species of SRB in the *Deltaproteobacteria* can promote the production of corrosive hydrogen sulfide from metallic sulfates (Kan et al., 2011). *Clostridia* was the second most abundant class in the majority of corrosion samples. This was similar observation to the results of a study of the composition of the bacterial community composition of biofilms from metal surfaces of an alkaline district heating system, and samples collected from water-flooded petroleum reservoirs (Kjeldsen et al., 2007; Tian et al., 2017). Some important SRB groups also belong to this class, for instance *Desulfotomaculum*. Some *Clostridia* produce acetic, butyric, or formic acids so that their presence may also lead to corrosion (Broda et al., 2000). Some are homoacetogens meaning that they convert carbon dioxide and hydrogen into acetate and propionate (Boga and Brune, 2003). The third most abundant class was the *Gammaproteobacteria*. Dang et al. (2011) reported that *Gammaproteobacteria* (mainly *Alteromonadales* and *Oceanospirillales*) are pioneer and long term surface colonizers, and can also contribute to the initiation and on-going development of biofilms. There are some other classes that were identified by this study that are known to contribute to steel corrosion, for instance *Epsilonproteobacteria* and *Zetaproteobacteria* (Dang et al., 2011; McBeth et al., 2011). Related research showed that *Epsilonproteobacteria* were the possible cause of microbial corrosion in pipelines injected with bisulfite (An et al., 2015).

At the genus level, three SRBs genera, *Desulfovibrio* spp., *Desulfotomaculum* spp. and *Desulfobacter* spp. formed a large proportion of the bacterial communities that were analyzed in this study. *Desulfovibrio* spp. were the most abundant. This complies with the data from a study of the corrosive marine biofilms of carbon steels immersed in seawater for 8 months (Bermont-Bouis et al., 2007). *Desulfovibrio* was also the most abundant bacterial genus in corrosion samples from oil pipelines in the Southeast of Mexico (Zhang and Fang, 2001; Neria-González et al., 2006; Vigneron et al., 2016). This agrees with previous studies that show that this genus is often the main cause of bacteria related corrosion (Miranda et al., 2006; Ilhan-Sungur et al., 2007). Members of genus *Desulfovibrio* are metabolically diverse and can reduce iron sulfate and, with hydrogen, produce H_2_S (Dinh et al., 2004). Significantly, the pH of an aquatic environment is modified by the presence of H_2_S, leading to the generation of a corrosion product, FeS in the presence of iron. The steel corrosion capacity of *Desulfovibrio* spp., such as *D. vulgaris* (Zhang et al., 2015, 2016), *D. alaskensis* (Wikieł et al., 2014) and *D. desulfuricans* (Lopes et al., 2006) has been extensively studied in laboratory experiments. Different mechanisms of corrosion development caused by *Desulfovibrio* spp. have been described and show that members of this genus have a interact with *Clostridium* species (Zhang and Fang, 2001), which was the second most abundant bacterial genus in sample SE4.

*Desulfotomaculum* spp. (the second most abundant genus) is a gram-positive SRB and is thermophilic. It plays an important role in MIC (Cetin et al., 2007) by accelerating cathodic depolarization and decelerating anodic depolarization (Cetin and Aksu, 2009). The ability of members of this genus to corrode steel has been studied extensively in laboratory experiments: *D. nigrificans* (Mystkowska et al., 2015), *D. orientis* (Ren and Wood, 2004), and *D. kuznetsovii* (Anandkumar et al., 2015). Members of this genus are usually associated with oil, and have been isolated from the crude oil field, oil production wells, or even the cooling towers of a petroleum refinery (Cetin et al., 2007; Cetin and Aksu, 2009; Anandkumar et al., 2015). *Desulfobacter* spp., is a mesophilic, gram-negative genus with an oval morphology in the marine environment. Its ability to oxidize acetic acid is a characteristic (Widdel, 1988). It can also reduce organic substrates to CO_2_ in a strictly anaerobic environment. However, the roles that *Desulfobacter* spp. play in steel corrosion are still unknown. Further study of its corrosive properties are needed. Some bacteria were found to inhibit MIC by the formation of a biofilm on the surface of steel. They included gramicidin-producing *Bacillus brevis* (Nikolaev and Plakunov, 2007), although *Vibrio neocaledonicus* may have the highest known level of corrosion inhibition (Moradi et al., 2015a,b). They did not appear in our results, but this might mean that they were present but at levels that were too low for detection, or that they were present at higher levels but were not detectable by techniques we adopted.

### Comparative Analysis of Bacterial Community Composition

It is well-known that biofilm maturity significantly affects the bacterial communities of biofilms (Neria-González et al., 2006). The succession pattern of these bacterial communities is tied to the immersion time of the steel. The steel could be exposed to local acidification with a decrease in the redox potential over time. This might stabilize the conditions so that the anaerobes are better accommodated. Also, the increase in the local concentration of dissolved iron salts may affect the biofilm community. Although SRB were dominant in all of our samples, the bacterial community composition of samples immersed for 8 years was significantly different to that of the sample immersed for 6 months. The bacterial diversity of the 6-months sample was higher than that the others. This result was consistent with previous studies. Bermont-Bouis et al. (2007) reported that there was a big difference between the bacterial community composition of 8 months immersion samples and 1 month immersion samples. They found that SRB were also the dominant population in mature biofilms after an 8 months immersion, but *Vibrio* spp. (*Gammaproteobacteria*) was the main component in samples that had been immersed for 1 month (Bermont-Bouis et al., 2007). This may be because biofilms form in a consistent series of discrete steps, or as a time series, each being associated with a different bacterial community (Stoodley et al., 2002).

Oxygen is consumed throughout the formation of biofilms. For instance, members of the *Bacteroidetes* may contribute to a decrease the quantity of oxygen emitted: these bacteria degrade high-molecular weight organic matter (Kirchman, 2002). The reduction of oxygen in the biofilms generates the anaerobic environment, which is needed to induce SRB growth. Over time, an increasingly acidic and anaerobic environment develops, and this is believed to result in the succession of membership of the biofilm community. In this study, the bacterial diversity of the samples immersed for 6 months was higher than that of the other samples. We believe that the anaerobic environment formed after 8 years was more suitable for the growth of SRB than that of the samples that had been immersed for 6 months. This implies that the anaerobic environment formed after 8 years was clearly unsuitable for the aerobic bacteria (the early colonizers), which is the reason for there being a reduction in bacterial diversity over time. That SRB may be only a minor component at the initial stage of biofilms is supported by Dang et al. (2011). Earlier studies have shown that the anaerobic zone will form underneath the upper aerobic layer when it is 10–25 mm thick (Coulter and Russell, 1976). At that point, the biofilm is clearly composed of a complex consortium of aerobic and anaerobic bacteria (Baker et al., 2003; Zhang et al., 2003). In addition, the bacterial community composition of biofilms is also changed most at the beginning of immersion (Dang and Lovell, 2000; Lee et al., 2008).

The methods of analysis can also have an impact on our understanding of the structure of bacterial communities. *Proteobacteria* was the dominant group for all corrosion samples no matter what methods were used. But the numbers of phyla and genera obtained from the corrosion samples were influenced by the methodology. We obtained more than 50 phyla and 100 genera by high-throughput Illumina sequencing (**Figures 4, 8**): that is many more than have previously been detected (Luan et al., 2012; Chen et al., 2014). Equally important, the number of OTUs obtained by high-throughput sequencing was greater than had been revealed by the traditional methods (plating) and T-RFLP technique. 19,581 OTUs were found in the nine corrosion samples by high-throughput Illumina sequencing in this study, whereas only 64 OTUs and 24 OTUs were previously identified in corrosion samples by PCR-RFLP (Luan et al., 2012; Chen et al., 2014). The high-throughput Illumina sequencing method is clearly ideal because we were able to achieve in depth quantitative analyses of microbial communities (Bokulich and Mills, 2012; Mayo et al., 2014).

Many environmental factors can affect the composition of bacterial communities. In this study, the richness of immersed steel was related to the sea location. The average richness of 8-years immersion samples from Xiamen was numerically lower than that of Sanya, although the difference was not significant. Among the environmental factors, salinity, pH and temperature generally have a significant effect on the bacterial community composition. Parallel research has shown that saline water irrigation can change bacterial metabolic activities and community structures (Chen et al., 2017). Cell growth rate was inhibited by high salinity, but the viability and integrity of the bacterial membrane were increased (Kim and Chong, 2017). The functional structure of a bacterial community was significantly affected by pH (Joshi et al., 2017). However, in our study, there was little difference in salinity at the two locations (Sanya 33.97aaa and Xiamen 31.96aaa) and pH (Sanya 8.48 and 8.56). As the seawater temperature at Sanya (25.14°C) was higher than that of Xiamen (19.27°C), we speculate that temperature caused the small difference. Many studies have shown that temperature is a major influence on the composition of bacterial communities in the marine environment. The composition of the bacterial community of crude oil-contaminated marine sediments or seawaters were shown by Bargiela et al. (2015) and Meng et al. (2016) to be strongly linked to temperature. Even more important, studies have shown that temperature is also related to corrosion levels. In the aquatic system, temperature plays an important role in the changes of most biofilm parameters, and in their propagation and metabolism (Bott, 1996; Rao, 2010). In addition, the amount of bacteria (whether aerobic or anaerobic bacteria) in biofilms was also temperature dependant (Bott, 1996; Guo et al., 2006). It has been reported that the amount of bacteria in the rust layer of immersed carbon steel in Yulin station was more abundant than that of Qingdao station because of the different temperature (Guo et al., 2006). In this study, the seawater temperature at Sanya was nearly 5°C higher than at Xiamen. The temperature of the Sanya coast was much more appropriate for the growth of bacteria. The degree of corrosion was enhanced by the presence of many more large fouling organisms in the warmer water. This resulted in the provision of higher levels of soluble nutrients provided by the decomposition of the other organisms. However, except for them, many other factors (like nutrients, dissolved oxygen and so on) could also influence the bacterial communities. The difference of the diversity was probably the result of integrated effects of the multiple factors. So far, it is hard to explain how the 6 months immersion sample (SOH) and 8 years immersion sample (SE4) were clustered in the same group in the multi-sample dendrogram. Our next step is to study the bacterial communities from samples from different substrates with a wider range of immersion times in a wider range of seawaters. This will greatly improve our knowledge of the relationships between environmental factors and bacterial community structure.

## Conclusion

The bacterial community composition of corrosion samples collected from rust layers of steel plates immersed in seawater for 6 months and 8 years at Sanya and Xiamen was revealed by means of Illumina MiSeq sequencing. We identified members of 13 phyla. *Proteobacteria*, *Firmicutes* and *Bacteroidetes* three dominated and accounted for nearly 89.03% of the total. *Desulfovibrio* spp., *Desulfotomaculum* spp. and *Desulfobacter* spp. were the core genera. The bacterial diversity from steel plate that has been immersed for 6 months was significantly higher than that taken from plates that had been immersed for 8 years. The average richness of biofilms removed from steel plates immersed for 8 years from Sanya was numerically but not significantly higher in similar samples taken from Xiamen at the same time. We identified bacteria that had not previously been found in this niche, although we do not know if they are involved in the corrosion of steel.

## Author Contributions

The article and experiment done by XL, YL took part in the experiment, experimental design done by JD and HX. The other authors took part in the sample collection.

## Conflict of Interest Statement

The authors declare that the research was conducted in the absence of any commercial or financial relationships that could be construed as a potential conflict of interest.

## Footnotes

1 http://drive5.com/uparse/

2 http://rdp.cme.msu.edu/

3 http://www.mothur.org/wiki/Schloss_SOP\#Alpha_diversity
